# Individual and Additive Effects of Insecticide and Mating Disruption in Integrated Management of Navel Orangeworm in Almonds

**DOI:** 10.3390/insects12020188

**Published:** 2021-02-22

**Authors:** Bradley S. Higbee, Charles S. Burks

**Affiliations:** 1 Trécé Inc., Adair, OK 74330, USA; 2 USDA, Agricultural Research Service, San Joaquin Valley Agricultural Sciences Center, 9611 South Riverbend Avenue, Parlier, CA 93648, USA; charles.burks@usda.gov

**Keywords:** mating disruption, navel orangeworm, *Amyelois transitella*, almond, integrated pest management

## Abstract

**Simple Summary:**

Mating disruption is an increasingly important part of pest management for the navel orangeworm *Amyelois transitella*. Industry groups have long supported mating disruption research and development with the divergent objectives of both minimizing damage from this key pest and reducing insecticide used on these crops. It is therefore important to know whether the benefits of mating disruption and insecticide are additive or, alternatively, if using both together provides no additional benefit over either alone. Ten years of data from research trials in a large commercial almond orchard found that the benefits of mating disruption are generally additive with lower damage if both are used together than either alone. Substantial year-to-year variability in navel orangeworm damage was also evident, even with stringent management. These findings indicate that the combination of mating disruption and insecticide can reduce the impact of navel orangeworm damage on the almond industry. Further improvements in monitoring and predictions of navel orangeworm abundance and damage are necessary for mating disruption to effectively contribute to the industry goal of reduction of insecticide use by 25%.

**Abstract:**

Damage from *Amyelois transitella*, a key pest of almonds in California, is managed by destruction of overwintering hosts, timely harvest, and insecticides. Mating disruption has been an increasingly frequent addition to these management tools. Efficacy of mating disruption for control of navel orangeworm damage has been demonstrated in experiments that included control plots not treated with either mating disruption or insecticide. However, the navel orangeworm flies much farther than many orchard pests, so large plots of an expensive crop are required for such research. A large almond orchard was subdivided into replicate blocks of 96 to 224 ha and used to compare harvest damage from navel orangeworm in almonds treated with both mating disruption and insecticide, or with either alone. Regression of navel orangeworm damage in researcher-collected harvest samples from the interior and center of management blocks on damage in huller samples found good correlation for both and supported previous assumptions that huller samples underreport navel orangeworm damage. Blocks treated with both mating disruption and insecticide had lower damage than those treated with either alone in 9 of the 10 years examined. Use of insecticide had a stronger impact than doubling the dispenser rate from 2.5 to 5 per ha, and long-term comparisons of relative navel orangeworm damage to earlier- and later-harvested varieties revealed greater variation than previously demonstrated. These findings are an economically important confirmation of trade-offs in economic management of this critical pest. Additional monitoring tools and research tactics will be necessary to fulfill the potential of mating disruption to reduce insecticide use for navel orangeworm.

## 1. Introduction

Mating disruption [1,2,3,4,5] is an increasingly important tool in integrated pest management (IPM) [6,7] and area-wide control of insect pests. It is used primarily against lepidopteran pests, although there are examples of mating disruption for control of Hemiptera [8,9], Coleoptera [10,11,12], and Hymenoptera [13]. Historically, synthetic pheromones and dispenser systems have been expensive [14,15]. Mating disruption use is most widespread in protection of high-value commodities such as horticultural crops, or in programs for management of invasive pests on public lands or across entire jurisdictions where management tactics are determined by policy objectives rather than cost-return criteria [5]. In some cases, mating disruption is used to reduce insecticide input and achieve the IPM goal of controlling pests with the least non-target impact, and in other cases, it is used with insecticides to achieve another IPM goal of maintaining economic sustainability. The degree of efficacy of mating disruption and the precise mechanisms by which it works varies with the target pest and the dispensing system, so the degree to which mating disruption is used to reduce insecticides vs. the degree to which it is used to reduce environmental impact varies with particular situations.

Mating disruption has become an increasingly prominent part of pest management for the navel orangeworm *Amyelois transitella* (Walker) (Lepidoptera: Pyralidae) [16]. The navel orangeworm is the principal pest of almonds and pistachios, and is an important pest of walnuts [16]. The area planted in each of these crops has increased substantially in the last 20 years [16]. Biological features of the navel orangeworm important to its pest status include its wide host range, its multivoltine life history, and a strong dispersal capacity [17,18,19,20]. The navel orangeworm directly attacks fruit, making it economically destructive. This polyphagous pest depends on host vulnerability for larval entry through lesions from disease, attack by another insect pest, or increased exposure of fruit with maturity [16]. Its robust dispersal capacity allows the navel orangeworm to move between orchards of different host crops, and its multivoltine nature and wide host range allows the navel orangeworm to amplify in one crop then go to another as earlier-maturing crops (e.g., almonds) are harvested and later-maturing crops (e.g., pistachios and walnuts) become vulnerable [16]. The adult does not feed, and the larva remains in its host through pupation until adult eclosion [16]. The navel orangeworm overwinters in unharvested fruit remaining on trees or dropped to the ground [16].

Integrated management of the navel orangeworm has long been based on the biology of the host and the phenology of the crop [16]. The most abundant and economically valuable almond variety in California is Nonpareil, which has a thinner shell and matures earlier than other varieties [21]. Nonpareil, like most almond varieties, requires cross-pollination with a different variety for optimal fruit set and so it is grown in orchards with multiple varieties [22]. Most of the pollinizer varieties planted alongside Nonpareil have a harder shell and a later harvest date, so they are less vulnerable to navel orangeworm but can be challenged by greater abundance [21,22]. Almonds in California generally flower in February and leaf out immediately afterward. For Nonpareil, hull split and vulnerability in healthy fruit generally begin in mid-June to early July, and harvest maturity is attained around six weeks later. The pollinizer varieties are typically three to four weeks behind in hull split and harvest maturity relative to Nonpareil. Almonds are harvested by shaking the nuts onto the ground. They are allowed to dry for several days, gathered into windrows on the ground, and taken to a huller for processing. Navel orangeworm from the overwintering generation are reproductively active in April and May and depend on almonds left from the previous year for a host. These overwintered navel orangeworm are referred to as first flight [23,24,25,26,27,28]. Their progenies form a second flight, which emerge in June around the time of Nonpareil hull split. The progeny of this second flight can develop faster because of better host quality, and the resulting third flight typically arrives in August around the time of Nonpareil harvest. A fourth flight typically arrives in September, around the time of harvest of the pollinizer varieties [16]. 

The pest management strategy based on this pest biology and host phenology relies most fundamentally on cultural practices of sanitation (rigorous removal and destruction of fruit left after harvest) and timely harvest [16,19]. If these tactics are not sufficient to keep navel orangeworm damage below an acceptable threshold, then insecticide treatments are also used [29,30,31,32,33]. Mating disruption for navel orangeworm has most often been used in addition to insecticide rather than as a replacement for it [34,35]. However, there is potential for reduced insecticide use.

The insecticides currently used most often for navel orangeworm include methoxyfenozide, chlorantraniliprole, various pyrethroids, and spinetoram [16]. All are targeted to eggs and/or neonates. Although the pyrethroids have had historically more activity than the others against adults, resistance has been documented (BH, unpublished data, and Niu et al. [36,37]). The time period when these products are most effective is the period immediately after the initiation of Nonpareil hull split [38]. The second most important time is a second preharvest application sometime between two weeks after the hull split application and the last possible application point before the preharvest interval [38]. These are both targeted against the second and third flights. In some cases, an application is made in April or May targeting first flight [38]. Applications also occasionally target the third flight in the period between the Nonpareil and pollinizer harvests, but often this is not done because of the complexity of coordinating the restricted access interval and other activities necessary during the harvest period. Use of more selective insecticides like methoxyfenozide or chlorantraniliprole is encouraged earlier in the season because these have a narrower spectrum of activity and are less likely to kill natural enemies that prevent defoliation by web-spinning mites. Decisions about the number of insecticide applications tend to be based on previous history and current crop prices. Monitoring assists in timing of insecticide applications, but predicting navel orangeworm damage based on in-season monitoring remains an ongoing challenge [39]. A further challenge to insecticide control results from the requirement that insecticide residue coverage prevents the larva entering the host where it is therefore sheltered from further exposure.

Currently, the most well-established formulation for mating disruption for navel orangeworm uses aerosol dispensers [40,41,42,43,44]. Peer-reviewed studies have also demonstrated efficacy for a hand-applied meso-dispenser formulation based on polyvinylchloride emitters [35]. Experimental formulations based on a more complete and attractive pheromone blend suppress males in pheromone traps more effectively than a single-component formulation, but all commercial formulations still use the single-component blend because of economic and regulatory considerations [43]. Mating disruption mechanisms are broadly categorized as competitive (the male interacts with the dispenser) or non-competitive (the male is made unresponsive to females without interacting directly with dispensers). The mechanism seems to be a hypothesized hybrid which initially involves attraction to the dispenser but then makes males unresponsive to females without continued interaction with the dispenser [2,15,45]. Like a purely non-competitive mechanism, the hybrid mechanism is less density dependent than competitive mechanisms [2,15,45]. Mating disruption for navel orangeworm provides the greater economic return with greater pressure within a range from moderate to high baseline damage [35]. 

Here, we present the damage data from ongoing mating disruption trials at a commercial almond site between 2006 and 2015 near the town of Lost Hills, CA. Methods that have been used to improve cost-effectiveness of aerosol mating disruption include limiting the part of the field season during which it is used, limiting the amount of pheromone loaded in each dispenser, and limiting the number of aerosol dispensers per ha. Previous studies analyzed the data from this and another site between 2009 and 2015 to examine the association of various monitoring methods with subsequent navel orangeworm damage, and to examine the relationship between variety composition and damage in these varieties [22,39]. In this paper, the Lost Hills data are analyzed using the randomized complete block design with which this site was arranged to compare navel orangeworm damage between plots treated with mating disruption alone, insecticide alone, or both. This long-term data set is used to examine effects of year-to-year variation on outcomes of management strategies for the navel orangeworm, and also year-to-year variation on relative impact of navel orangeworm on two major almond varieties, Nonpareil and Monterey.

## 2. Materials and Methods 

### 2.1. Site and Plot Arrangement

**Figure 1:**
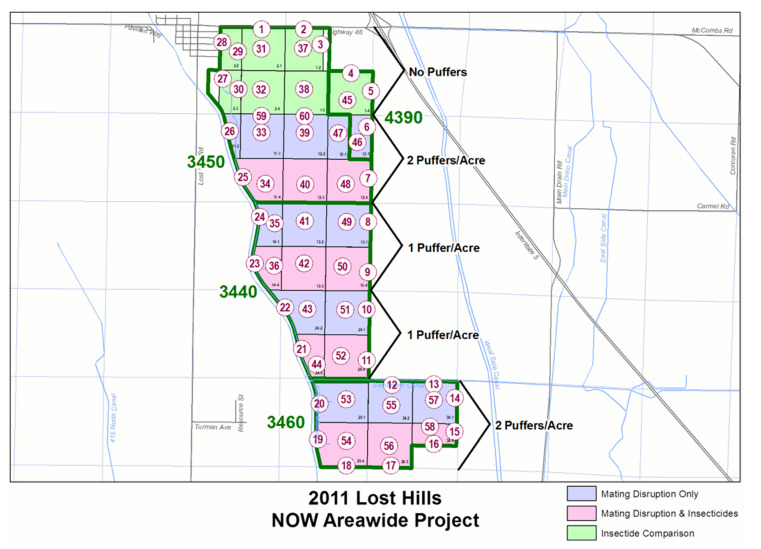
Representative map for treatment areas (blocks) in a commercial almond orchard in 2011. The two blocks with sites 4, 5, 6, 45, and 46 (upper right portion of map) are pistachio orchards and not included in this study.

Trials were performed on 971 hectares of almond trees from 2006 to 2015 at the Lost Hills Ranch, planted in 1990 and 1993, owned and operated by Wonderful Orchards (formerly Paramount Farming). From 2008 to 2012, these were part of a USDA Agricultural Research Service area-wide integrated pest management project to improve navel orangeworm management [22,46]. General features of this site are illustrated by a plot map from 2011 (Figure 1). The basic management units at this site were partial or complete 54 ha (160 ace) quarter-sections [47]. Collections of these quarter-sections were referred to as ranches. In this figure, almond ranches (delineated by heavier green lines) include 3450, 3440, and 3460. The pistachio ranch 4390 was not included in the current analysis. East-west tiers of quarter-sections served as replicate blocks. For example, in Ranch 3450, the third and fourth tiers from the top (respectively, purple and pink) served as replicate blocks for a mating disruption treatment with and without insecticide. All blocks were subject to an intensive sanitation regime that combined machine and hand removal of nuts from trees and flail mowing of residue following harvest to eliminate mummies from managed areas. This resulted in 5–10 mummies/tree on the ground and less than 0.3 mummies/tree in the canopy [19]. All blocks had Nonpareil almonds. The most common pollinizer variety was Monterey. Other pollinizer varieties included Butte, Carmel, Fritz, Mission, Price, Ruby, and Wood Colony.

Four different experiments were performed at this site from 2006 to 2015: (1) An experiment in 2006 and 2007 comparing mating disruption at label rate used alone, insecticide used alone, and both together; (2) an experiment from 2008 to 2011 examining aerosol mating disruption dispensers per hectare; (3) an experiment from 2012 to 2014 examining different concentrations of pheromone emitted from dispensers at a fixed spatial density; and (4) an experiment in 2015 examining the date that mating disruption started. The latter three experiments were elaborations of the initial experiment; thus, they were amenable to a common analysis.

### 2.2. Evaluation of Navel Orangeworm Damage

Damage to almonds by navel orangeworm was evaluated by two methods, which we refer to here as windrow and huller. For research evaluation, windrow samples of approximately 500 nuts were gathered when each variety was harvested from the ground or windrow at each of the numbered monitoring sites in Figure 1. Sampled nuts were evaluated individually in the laboratory. Nuts were opened and scored for whether the kernel was damaged (navel orangeworm damage), and for whether there were navel orangeworm larvae associated with the hull in almonds with undamaged kernels (proportion of navel orangeworm infestation was the proportion of nuts with either kernel damage or infestation without kernel damage). The numbered sites corresponded to monitoring stations and most quarter-sections had a monitoring site on the edge of the orchard and one on the center. The monitoring sites on the edge were important for trapping and monitoring, but only the center sites were used for evaluation of effects of mating disruption and insecticide treatments.

Huller evaluation used methods similar to industry-standard practice. Windrowed almonds are picked up from windrows, placed in hopper trailers, and taken to a huller for initial processing. At the huller, after field debris has been removed and the kernels are removed from the hulls and shells, damage to kernels is evaluated from a 20 kg sample from each trailer as a basis of quality control and payment. In standard industry procedure, kernels are determined to be edible or non-edible, and non-edible kernels are noted by a broad range of damage categories. In the current study, a further assessment was made to determine if non-edible status was the result of navel orangeworm damage.

### 2.3. Individual and Additive Effects of Mating Disruption and Insecticide (2006 and 2007)

In 2006 and 2007, the treatments were: (1) aerosol mating disruption with 5 dispensers per ha (the first label rate [15,41,43]); (2) applications of the insecticide methoxyfenozide targeting the first and second navel orangeworm flights (Table S1); and (3) a combination of both of these treatments.

### 2.4. Mating Disruption Dispensers per Hectare (2008–2011)

Subsequent experiments examined more and less intensive forms of mating disruption with and without an insecticide treatment. From 2008 to 2011, aerosol mating disruption at two different dispenser densities, with or without insecticide, was compared to insecticide treatment alone. Treatments were thus: (1) insecticide treatment without mating disruption; (2) 2.5 mating disruption dispensers per ha without insecticide; (3) 2.5 mating disruption dispensers per ha with insecticide; (4) 5 mating disruption dispensers per ha without insecticide; and (5) 5 mating disruption dispensers per ha with insecticide. Mating disruption treatments used Suterra Checkmate Puffer NOW aerosol dispensers, each of which contained 3.8 g of the active ingredient (a.i.) (*Z*11,*Z*13)-hexadecadienal and releasing 0.38 mg every 15 min from 17:00 to 05:00 local time for a total of 18.24 mg per dispenser per night [15,41,43]. The two replicates of the no-mating disruption insecticide treatment were placed adjacent to each other and at either the north or south end of the site to minimize the effect of the mating disruption treatments on these no-mating disruption treatment blocks. Insecticide treatments for navel orangeworm consisted of two applications per year, approximately as described in the previous section (Table S1). 

### 2.5. Mating Disruption Active Ingredient Per Hectare (2012–2014)

An experiment from 2012 to 2014 examined aerosol mating disruption with or without insecticide in a manner similar to the previous experiment. However, all mating disruption blocks were treated using 5 dispenser per ha, using either the standard rate or half of the standard rate. Amount of a.i. per ha was varied by the amount of a.i. in the aerosol cannister (3.8 or 1.9 mg), and therefore 0.38 or 0.19 mg a.i. per emission and 91 or 45 mg a.i. per ha per night. Treatments were thus: (1) insecticide treatment without mating disruption; (2) 5 mating disruption dispensers per ha, each containing 1.9 mg a.i., without insecticide; (3) 5 mating disruption dispensers per ha, each containing 1.9 mg a.i. with insecticide; (4) 5 mating disruption dispensers per ha, each containing 3.8 mg a.i., without insecticide; and (5) 5 mating disruption dispensers per ha, each containing 3.8 mg a.i., with insecticide. In 2012, methoxyfenozide was applied in spring and at hull split, similar to the previous years. In 2013 and 2014, three applications were made against navel orangeworm, with the pyrethroid, bifenthrin, applied post-hullsplit, and prior to the Nonpareil harvest (Table S2). 

### 2.6. Time of Start of Mating Disruption (2015)

The variable for aerosol mating disruption for 2105 was the time that mating disruption began: either early season (17 March, 336 NOW degree-days from 1 January) or normal deployment (13 April, 577 NOW degree-days from 1 January) of mating disruption in combination with conventional treatment. Treatments were thus: (1) insecticide treatment without mating disruption; (2) the standard mating disruption timing without insecticide; (3) the standard mating disruption timing with insecticide; (4) the early mating disruption timing without insecticide; and (5) the early mating disruption timing with insecticide. The insecticide regime in 2015 was similar to 2013 and 2014 (Table S2). Mating disruption trials at this site were discontinued after a single year of this experiment. 

### 2.7. Data Analysis

Data were processed and plotted using R 4.0 [48]. Correlation, linear regression, and nonparametric analysis was performed in R, and generalized linear mixed model (GLMM) analysis was conducted using the SAS system [49]. An initial analysis compared damage effects between edge windrow samples, interior windrow samples, and huller samples. In this case, the experimental units were the 65 ha “sections” rather than the tiers that formed replicate blocks. This was done because the sections were the smallest unit for which there were independent data for the huller samples. The edge and interior windrow samples were aggregated (there were not edge samples for all sections, since some were bounded on all sides by other sections). Damage over 10 years was compared between three types of treatment (mating disruption, insecticide, or both) for the three types of samples using the non-parametric Kruskal–Wallis ANOVA followed by the Dunn post hoc test, with the holm procedure for means separation. In addition, ordinary least squares regression was used to compare percent *A. transitella* damage in interior and edge windrow samples with *A. transitella* damage in huller samples.

A subsequent analysis compared damage across all 10 years, based on the fact that the general treatment structure was used in 2008 to 2015 was an overlay on the three-way comparison in 2006 and 2007. Based on the initial analysis, the interior windrow samples were used for this analysis. Damage and total nuts examined were pooled across the tier that formed replicate blocks (Figure 1), and analyzed as using a GLMM (PROC GLIMMIX) with a binomial error distribution, and Kenward–Roger degrees of freedom [50]. The treatment (insecticide, mating disruption, or both) was a fixed factor, and the year and tier (replicate block) were random factors. The binomial samples were based on a mean sample size of 5336 (range 1072 to 12,753).

In addition to analyzing the entire 10-year data set, experimental variations were analyzed separately. The same fixed and random independent variables were used for data from 2006 and 2007. For the experiments from 2008–2011, 2012, 2014, and 2015, data from the insecticide-only plots were set aside and the experiments were analyzed as a 2 × 2 factorial design with one factor representing two different intensities of mating disruption, and the other factor representing presence or absence of insecticide treatments. 

Damage in the varieties Nonpareil and Monterey was compared only in the plots treated with insecticides and not mating disruption, in order to minimize pest management treatment as a confounding factor in this comparison over the 10 years. Comparisons were made using the windrow interior samples. In addition, navel orangeworm degree-days from January 1 were calculated using the UC IPM degree-day calculator [51] and data from the Lost Hills (Kern County) California Irrigation Management Information System (CIMIS) site [52], and degree-day accumulation on 15 June was compared between years in which Nonpareil had more damage than Monterey and years in which the converse was true.

## 3. Results

### 3.1. Comparison of Huller and Windrow Samples

Damage in all almond varieties over the 10-year study differed based on treatment type and the type of sample used to evaluate the treatment (Table 1). In all cases, damage over the 10-year period was numerically higher in mating disruption plots than in plots treated only with insecticide, but this difference was not significant (*p* > 0.05) in huller samples and in interior windrow samples collected by researchers. Regression revealed a significant association of the internal and the edge windrow samples with the huller sample (Table 2). These comparisons are based on all blocks with both edge and internal collection sites. Based on these observations, the windrow internal samples were used for subsequent analysis because the raw data were more uniform than the huller reports, and more suitable for statistical analysis (i.e., direct quantification of damage and total sample size was preserved).

### 3.2. Comparison of Overall Damage over the 10-Year Study

**Figure 2:**
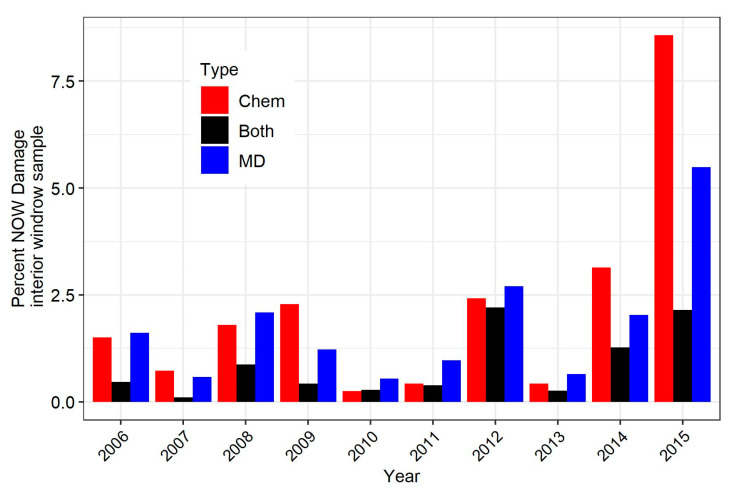
Mean navel orangeworm damage for all almond varieties for 2006 to 2015, as determined from interior windrow samples. The insecticide (Chem) indicates plots that received insecticide but not mating disruption; the mating disruption (MD) treatment category includes all mating disruption treatments when used without insecticides, and the Both treatment category includes all mating disruption treatments when used with insecticide.

Interior windrow samples from all blocks (aggregated into replicate tiers) were used for a more comprehensive comparison of damage over the period of the study. Over the 10 years, navel orangeworm damage was different among treatments (*F*_2,91.8_ = 14.06, *p* < 0.0001). Plots treated with both insecticide and mating disruption had significantly less damage than those treated with either insecticide alone or mating disruption alone, while there was no significant different among the latter two treatments (Table 3). A graph of damage based on interior windrow samples revealed that it was numerically lower in the plots treated with both mating disruption and insecticide compared to those treated with only mating disruption or only insecticide in 9 of the 10 years examined (Figure 2). There was substantial variation in navel orangeworm damage with the average across all treatments ranging from 0.36% in 2010 to 5.4% in 2015. 

### 3.3. Comparison of Damage in Specific Experiments

Analysis of data from the first two years, in which there were only three treatments, revealed similar trends to the 10-year data set. There were significant differences among the treatments (*F*_2,7.05_ = 6.59, *p* = 0.02), and the mating disruption and insecticide treatments were not different while the combined treatment had significantly less damage (Table 4).

→ For the experiment from 2008 to 2011, there were numerical differences among all levels of the factorial comparison of 2.5 or 5 mating disruption dispensers with or without insecticide (Table 5). The GLMM analysis of fixed effects revealed significant effects due to insecticide (*F*_1,13.75_ = 11.34, *p* = 0.0047), not quite significant effects due to dispenser density (*F*_1,13.76_ = 3.33, *p* = 0.0896), and no significant interaction (*F*_1,24.59_ = 0.42, *p* = 0.52).
→ For the experiment from 2012 to 2014, percent navel orangeworm damage in mating disruption plots was, respectively, 1.6 ± 0.57 and 1.1 ± 0.39 for half and full label concentration mating disruption from 5 dispensers per ha in the absence of insecticide treatments. In the presence of insecticide, these figures were, respectively, 0.9 ± 0.61 and 0.7 ± 0.38 percent damage. There were no significant effects from either insecticide or mating disruption release rate, and the interaction was not significant (*p* > 0.1).

The analysis also found no significant effects for the 1-year experiment at the time of the start of mating disruption, conducted in 2015. Percent damage in mating disruption plots was, respectively, 10.3 ± 9.4 and 3.0 ± 0.93 for the early and standard mating disruption start times in the absence of insecticide treatments. In the presence of insecticide, these figures were, respectively, 2.6 ± 0.44 and 1.9 ± 1.31 percent damage. 

### 3.4. Relationship Between Damage in Early-Harvested Nonpareil and a Later Harvested Pollinizer Variety

**Figure 3:**
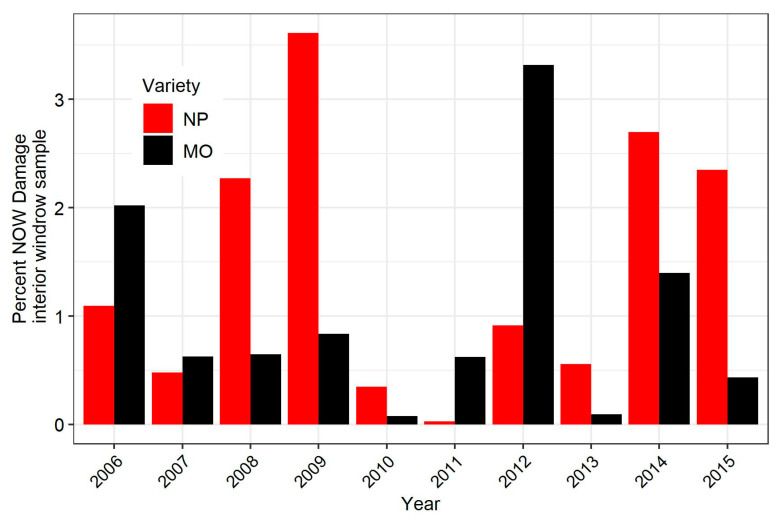
Percent navel orangeworm damage by year in Nonpareil and Monterey almonds in plots treated only with insecticide.

Over a 10-year period, the mean navel orangeworm damage in the insecticide-treated plots was numerically higher in Nonpareil almonds (1.4 ± 0.38) than in Monterey almonds (1.0 ± 0.32). This difference was not statistically significant (Welch unequal variance t-test, t = 0.86, df = 17.402, *p* = 0.40). A year-by-year graph of damage shows that the relative damage between the varieties was highly variable, and in 4 of the 10 years, damage was higher in Monterey than in Nonpareil (Figure 3). The mean navel orangeworm degree-day (°C) accumulation on June 15 was 638 ± 57 for the 4 years when Monterey damage was higher than Nonpareil damage, and 726 ± 50 for the 6 years when Nonpareil damage was higher. This difference was not significant (Welch unequal variance t-test, t = −1.17, df = 6.915, *p* = 0.28).

## 4. Discussion

In this study, windrow samples taken from the interior of the block and the edges were both strongly associated with percent damage from huller samples. Huller samples provide a more immediately relevant assessment of treatment effects on damage to almonds compared to windrow samples more typically used in research projects [15,35,41,43,45]. There are, however, trade-offs between the two approaches. Previously, industry and extension personnel have estimated that windrow samples have about twice the navel orangeworm infestation and damage compared to huller samples [35]. The difference between these two sampling methods is attributed to loss of damaged almonds between the two sampling points [35]. Air legs at various points in the harvest and processing pathway separate almonds from lighter field trash. After extensive feeding, damaged almonds can partition with this field trash; therefore, yield loss from navel orangeworm can be underestimated by the yield data typically received by a grower from a huller-processer. In the present study, we were able to take the extra step of further examination of damaged kernels from the huller to determine which were damaged by navel orangeworm. That option is rarely available to researchers. However, in addition to capturing more of the true loss to navel orangeworm damage, the researcher-gathered windrow samples have the advantages of being collected for the purpose of analysis. Unlike huller reports, which provide a percent damage for a large but imprecisely known sample, the windrow data provide integers representing nut damages and nuts examined, and therefore work better with analysis by generalized linear models. In contrast to the 2× estimates used in previous studies, we found that windrow samples taken from the interior of the orchard has 1.42× the damage of huller samples. 

Navel orangeworm damage in edge samples also correlated well with huller samples, albeit not as well as internal windrow samples. Navel orangeworm damage in the edge samples was higher for all treatments. This higher damage is likely due both to mated females immigrating from sources of higher abundance outside the study site, and to faster maturation of almonds on the edges of the orchard. This faster maturation makes infestation of the almonds in the outside portion of the orchard a leading indicator of infestation in the orchard. Sampling edge site has therefore been tested as part of a multi-factor monitoring program to improve prediction of damage from navel orangeworm [39]. The association reported here of navel orangeworm damage with huller damage further supports the utility of this multi-factor monitoring approach. 

Because mating disruption is often used with high value crops, studies of its efficacy are often done as an “overlay” in which plots treated with mating disruption and insecticide are compared to plots treated with insecticide alone [53]. Early mating disruption studies for navel orangeworm were unusual in using large untreated control plots without insecticide [41]. Subsequent studies have used the overlay approach [15,35]. The value of the crop at Lost Hills (10s of millions of dollars annually) precluded further untreated controls. The present approach allowed a valuable comparison of the contributions of an established and a new technology in integrated management of navel orangeworm. 

Analysis of data for the first three years, when typical use of mating disruption and insecticide was compared using either alone or both together, found consistently similar damage between mating disruption and insecticide, and significant reduction when both were used together (Table 4, Figure 2). Broad analysis found this pattern through the 10-year study (Table 3), but with much variation (Figure 2). Mechanistically, the superior performance of mating disruption and insecticide together to either alone is plausible because they act at different points in the pest’s lifecycle. Mating disruption acts against adults, reducing or preventing fertility [2,4]. Methoxyfenozide, the predominant insecticide in this study, primarily kills eggs and neonates [29]. Navel orangeworm eggs are laid on almonds close to the suture, and once the neonate larva enters the almond, it is no longer exposed to insecticide. 

Experiments from 2008 to 2015 sought to improve cost-effectiveness of the aerosol dispensers. Aerosol dispensers initially proved to perform better than the hand-applied devices then tested [41]. Subsequent data indicates similar crop protection from several aerosol formulations now marketed, and from a meso-dispenser formulation [35]. Mechanisms of mating disruption invoked by the aerosol formulations [15,45] are likely broadly similar to those invoked by the meso-dispenser formulation (CB, unpublished data). Microencapsulated (sprayable) formulations are also commercially available, but do not offer the season-long suppression provided by the aerosol and meso-dispenser products (BH, unpublished data). The data from 2008 to 2011, comparing 2.5 or 5 dispensers per acre, found a marginally significant effect from the number of dispensers, but a stronger effect from using insecticide in addition to 2.5 dispensers per acre (Table 5). 

The amount and year-to-year variation in the huller damage from navel orangeworm illustrates additional difficulties in obtaining full IPM benefits from mating disruption. Another recent study found between 0.9% and 1.1% huller damage as a breakpoint above which mating disruption for navel orangeworm in almonds increases grower return [35]. That analysis was based on the premium and penalty of another large almond processor, and may not be entirely applicable to a vertically integrated company such as Wonderful Farming. Processors and industry groups like the Almond Board of California (ABC) [54] tend to be concerned with providing the cleanest product possible in order to maintain the broadest market possible for almonds, and they may receive benefits from extra pest management input that would not accrue to a grower selling to a major processor. It is also illustrative that variable and occasionally high damage occurred despite overall favorable conditions for control of navel orangeworm. The orchards were managed by a well-capitalized company, and there was an ongoing commitment to orchard sanitation (winter removal and destruction of unharvested almonds) to a far higher degree than is common practice [19]. 

The aforementioned observations are consistent with the recent suggestion that mating disruption for navel orangeworm is a prudent insurance against high damage [35]. However, these observations also demonstrate the difficulty of fully realizing the potential for mating disruption to reduce insecticide input, such as the 25% reduction called for by ABC between 2020 and 2025 [54], and realizing such reduction will require improved monitoring methods, and greater adoption and confidence in such measures. Data from this and other commercial orchards over part of this period were used to determine which components of a multipart monitoring system best predicted damage [39]. That study found that pre-harvest sampling of almonds and trapping for females provided the best available prediction of damage, with an *r*^2^ of approximately 0.5. Alternative attractants provide improved detection of navel orangeworm and are less impacted by mating disruption [42,55,56,57]. It is unclear, however, whether captures baited with these attractants which capture both sexes in traps [42] are as directly related to damage as the female traps in the previously mentioned study, and it appears there may be a trade-off between prediction power and detection sensitivity. Further, monitoring gains to provide greater confidence in the ability to base insecticide applications on in-season data may come from improved female attractants and trapping systems, possibly aided by improvements in trap automation and information [58]. 

The year-to-year variation in relative damage in Nonpareil further illustrates the complexity of navel orangeworm damage. Monterey is widely planted, and was the most prevalent variety in the current study site after Nonpareil. Nonpareil is the most commercially valuable almond variety, and has the poorest shell seal and therefore is most exposed to navel orangeworm [21]. Monterey has a much tighter shell seal, and is therefore thought of as less susceptible to navel orangeworm [21]. However, Monterey matures six weeks later than Nonpareil and, therefore, navel orangeworm populations are often in another generation and more abundant by the time Monterey is susceptible. This might be why, in a previous three-year study that found a negative correlation between shell seal and navel orangeworm infestation across varieties, Nonpareil and Monterey had similar damage [21]. The more long-term data from this study indicate greater variation than observed in this previous three-year study [21]. The hypothesis that a tighter shell seal in Monterey is offset by greater navel orangeworm abundance suggests that greater damage in Monterey than Nonpareil might come in cooler years, when Nonpareil would be less exposed to navel orangeworm. The comparison of degree-day accumulation at 15 June suggests that degree-day accumulation does not predict relative damage between Nonpareil and Monterey. It is possible that phenology of the nut is as important to damage patterns across varieties as phenology of the moth: for example, years in which poor conditions at bloom and pollination (in February) might impact Monterey more than Nonpareil. Such conditions cause more uneven maturation and delay harvest, therefore causing greater exposure. This hypothetical explanation is speculative, but illustrates that research to improve prediction of navel orangeworm damage needs to consider both the phenology of the navel orangeworm and that of the host. 

## 5. Conclusions

Navel orangeworm damage trends in this 10-year case study showed a consistent trend of lower damage in almond plots treated with both insecticide for navel orangeworm and mating disruption compared to either alone. This study also provided a more quantitative estimate of the relationship between field and processor damage from navel orangeworm, confirming that the processor data understate loss from navel orangeworm. Variation from year to year in the relative navel orangeworm damage between two widely planted varieties with different maturities demonstrates the importance of protecting all varieties, and considering all varieties when comparing tactics for reduction of navel orangeworm damage. Year-to-year variation in navel orangeworm damage despite stringent management illustrates the challenge in taking mating disruption for navel orangeworm from a tool to lower risk of navel orangeworm damage to a tool to advance the industry goal of lowering insecticide input. 

## Figures and Tables

**Table 1:** Median damage (percent) of all almond varieties by treatment type over 10 years.

Treatment Type	Huller	Windrow (Interior)	Windrow (Edge)
Mating disruption only	0.61a	0.91a	2.22a
Insecticide only	0.56a	0.73a	1.58ab
Both	0.3b	0.37b	1.01b

Medians in the same column followed by different letters are significantly different (Kruskal–Wallis ANOVA, experiment-wise *p* < 0.05).

**Table 2:** Linear regression of percent damage of windrow samples on huller samples.

	Windrow (Interior)	Windrow (Edge)
Intercept	−0.01	0.69 ***
Slope	1.42 ***	1.75 ***
Adjusted *r*^2^	0.69	0.59

*** *p* < 0.001.

**Table 3:** Percent navel orangeworm damage (mean ± SE) from interior windrow samples, by insecticide and mating disruption treatment and across all varieties, 2006–2015.

Treatment	Percent NOW Damage
Insecticide only	1.9 ± 0.47a
Mating disruption only	1.8 ± 0.49a
Both insecticide and mating disruption	1.0 ± 0.18b

Means followed by different letters are significantly different (generalized linear mixed model (GLMM) with binomial distribution, *p* < 0.05).

**Table 4:** Percent navel orangeworm damage (mean ± SE) from interior windrow samples, by insecticide, and mating disruption treatment and across all varieties, 2006 and 2007.

Treatment	n (Replicate Block by Year)	Percent NOW Damage
Insecticide only	9	1.0 ± 0.24a
Mating disruption only	4	1.1 ± 0.32a
Both insecticide and mating disruption	3	0.4 ± 0.13b

Means followed by different letters are significantly different (GLMM with binomial distribution, *p* < 0.05).

**Table 5:** Percent navel orangeworm infestation (mean ± SE, n = 8) from windrow samples from Nonpareil and pooled pollinizer varieties by insecticide and mating disruption (MD) treatment, 2008–2011.

Mating Disruption Dispensers per ha	Without Insecticide	With Insecticide
2.5	1.67 ± 0.64	0.64 ± 0.20
5	0.93 ± 0.22	0.33 ± 0.09

The row-wise differences (insecticide effect) are significant (*p* < 0.05), the column-wise differences (dispensers per ha) are not quite significant (0.1 > *p* > 0.05), and the interaction is not significant ((*p* > 0.1) (GLMM with negative binomial distribution).

## Data Availability

The almond damage and navel orangeworm degree (°F) data used in this paper are available in a public repository (doi 10.5281/zenodo.4553809; see https://doi.org/10.5281/zenodo.4553809 accessed on 21 February 2021).

## Supplementary Materials

The following are available online at https://www.mdpi.com/2075-4450/12/2/188/s1, Table S1: Insecticides used in Lost Hills 2006–2011, Table S2: Insecticide applications in Lost Hills 2012–2015.
