# Supplementary material for: Individual and Additive Effects of Insecticide and Mating Disruption in Integrated Management of Navel Orangeworm in Almonds
Source: Insects. 2021 Feb 22;12(2):188. doi: 10.3390/insects12020188 (PMC7927001; doi:10.3390/insects12020188)
Supplement: Supplementary file 1 [file insects-12-00188-s001.pdf]

Table S1. Insecticides used in Lost Hills 2006-2011 – 2 puffer/ac vs 1 puffer/ac vs Insecticides comparisons. All Conv and MD+Conv blocks (CMD) were treated with insecticides for NOW. Essentially two spray of Intrepid/year. All applications were Intrepid @18oz/ac +16 oz exit, 200 gals/ac

2006 - 2 Intrepid appls. @18oz/ac +16 oz exit, 200 gals/ac<br>1st spray @ 150 DD 1st flight on April 24 – 28. + Abamectin + oil<br>2nd spray @ 250 DD 2nd flight in Mid- June was planned but actually applied July 5-7 @ 400 dd 2nd flight.
2007 -2 Intrepid appls @18oz/ac +16 oz exit, 200 gals/ac<br>1st on@ 450 DD 1st flight (May 3-5) + Abamectin + oil<br>2nd @ 325 DD 2nd flight (June 26).
2008 - 2 Intrepid applications @18oz/ac +16 oz exit, 200 gals/ac<br>1st on April 26-28 + Abamectin + oil<br>2nd on July 2-5 + 32 oz Fujimite R345
2009 - 2 Intrepid applications @18oz/ac +16 oz exit, 200 gals/ac<br>1st on April 13-15 + Abamectin + oil<br>2nd on June 26 – July 2 + 32 oz Fujimite R345
2010 - 2 Intrepid applications @18oz/ac +16 oz exit, 200 gals/ac<br>1st on April 26-28 + Abamectin + oil<br>2nd on July 6-12 + 32 oz Fujimite R345
2011 - 2 Intrepid applications @18oz/ac +16 oz exit, 200 gals/ac<br>1st on April 27-30 + Abamectin + oil<br>2nd on July 11-16 + 32 oz Fujimite R345

Table S2. Insecticide applications and their active ingredient (AI) in the orchard treatment areas (Conventional (C), and Conventional + Mating Disuprtion C+MD) targeting, or with an impact on, navel orangeworm (NOW) moths for growing seasons in 2012 through 2015.

Year	Target Pest	AI	AI /ha	Application Date	DD[1] from Jan 1
2012	NOW	Methoxyfenozide[2]	0.31 kg	5/9	344 (652)
2012	NOW	Methoxyfenozide[2]	0.31 kg	7/9	927 (1700)
2013	NOW	Methoxyfenozide[2]	0.31 kg	4/20	168 (335)
2013	NOW	Methoxyfenozide[2]	0.31 kg	6/28	814 (1498)
2013	NOW	Bifenthrin[3]	0.09 kg	7/29	1256 (2293)
2014	NOW	Methoxyfenozide[2]	0.43 kg	4/15	317 (602)
2014	NOW	Methoxyfenozide[2]	0.43 kg	6/27	969 (1777)
2014	NOW	Bifenthrin[3]	0.09 kg[4]	7/9	1413 (2575)
2015	Plant bugs	Lamdba-cyhalothrin[5]	0.045 kg	4/17	321 (609)
2015	NOW	Methoxyfenozide[2]	0.43 kg	6/26	930 (1706)
2015	NOW	Chlorantraniliprole[6]	0.11 kg	7/20	1247 (2276)

2015	NOW	Bifenthrin[7]	0.22 kg	7/20	1247 (2276)

[1]Degree days of navel orangeworm in Celsius (Fahrenheit).
[2]Intrepid 2 F, Dow AgroSciences, Indianapolis, IN, USA.
[3]Bifenture, United Phosphorus, Inc., King of Prussia, PA, USA.
[4]Sprayed alternate rows, 50% of orchard sprayed.
[5]Warrior II, Syngenta Crop Protection, LLC, Greensboro, NC, USA.
[6]Altacor, DuPont Crop Protection, Newark, DE, USA.
[7]Brigade WSB, FMC Corp. Agricultural Products Group, Philadelphia. PA, USA.
